# Recrystallization Behavior of Cold-Rolled AA5083 Microalloyed with 0.1 wt.% Sc and 0.08 wt.% Zr †

**DOI:** 10.3390/ma18081701

**Published:** 2025-04-09

**Authors:** Ahmed Y. Algendy, Paul Rometsch, X.-Grant Chen

**Affiliations:** 1 Department of Applied Science, University of Quebec at Chicoutimi, Saguenay, QC G7H 2B1, Canada; aalgendy@etu.uqac.ca; 2 Arvida Research and Development Center, Rio Tinto Aluminium, Saguenay, QC G7S 4K8, Canada; paul.rometsch@riotinto.com

**Keywords:** Al-Mg-Mn AA5083 alloy, Sc and Zr microalloying, recrystallization, microstructure, annealing temperature, Al_3_(Sc,Zr) precipitates

## Abstract

The influence of annealing temperature on the mechanical properties, microstructural evolution, and recrystallization behavior of AA5083 cold-rolled sheets with and without Sc/Zr microalloying was studied utilizing hardness tests, optical microscopy, electron backscatter diffraction, and transmission electron microscopy. The results show that a minor addition of Sc/Zr to the Al-Mg-Mn alloy can significantly improve the alloy strength and recrystallization resistance. Adding 0.1 wt.% Sc and 0.08 wt.% Zr raised the recrystallization temperature of heavily deformed sheets to 500 °C, which is 250 °C higher than for the Sc-free base alloy. The higher recrystallization resistance of the Sc-bearing alloy was mainly attributed to the presence of Al_3_(Sc,Zr) nanoparticles, which enhanced the Zener drag pressure and delayed recrystallization. Grain boundary strengthening effects at various annealing temperatures were estimated using a constitutive equation. This work revealed that grain structure change and the corresponding boundary strengthening effect are key factors governing alloy strength evolution during annealing.

## 1. Introduction

Al-Mg-Mn alloys are widely used in various industrial applications, particularly in the automotive, marine, and aerospace industries, due to their excellent combination of a high strength-to-weight ratio, corrosion resistance, formability, and weldability [1,2,3]. Microalloying with scandium (Sc) and zirconium (Zr) has been identified as a novel approach to enhance the overall mechanical performance of Al-Mg-Mn alloys [1,2,4,5,6]. Sc and Zr form coherent, thermally stable L1_2_-Al_3_(Sc,Zr) nanoparticles in the Al matrix during heat treatment, resulting in the improvement of room- and elevated-temperature mechanical properties, as well as recrystallization and corrosion resistance [2,4,5,6,7,8,9,10].

Recrystallization is a thermally activated process, in which deformed grains undergo nucleation, growth, and coalescence to form new equiaxed strain-free grains [11]. It plays a vital role in determining a material’s final microstructure and mechanical properties. The annealing temperature is a key parameter that can profoundly affect the recrystallization behavior and, consequently, the overall alloy performance [6,12,13,14]. Increasing the annealing temperature can promote dislocation polygonization and subgrain coalescence, accelerating the recrystallization process, lowering the alloy strength, and enhancing the ductility.

Commercial grades of Al-Mg-Mn alloys often exhibit limitations in recrystallization resistance, which can impact their final mechanical properties. Adding Sc and Zr to Al-Mg-Mn AA5083 alloys enhances their resistance to recrystallization and restrains grain growth during the annealing treatment [13,14,15]. Microalloying Sc forms fine L1_2_-Al_3_Sc nanoprecipitates that enhance alloy performance by impeding dislocation motion and grain boundary migration. However, these precipitates coarsen with increasing temperatures, reducing their effectiveness. The co-addition of Zr creates a Zr-enriched shell around the Al_3_Sc precipitates, forming thermally stable L1_2_-Al_3_(Sc,Zr) particles with better resistance to coarsening due to the lower diffusion coefficient of Zr. In addition, the core–shell configuration of Al_3_(Sc,Zr) precipitates minimizes lattice mismatch with the α-Al matrix, resulting in a strong coherent interface, thereby effectively enhancing strength and recrystallization resistance [6,16,17]. It is reported [17] that an Al–3Mg alloy was fully recrystallized after a 5 min of annealing at 360 °C, whereas adding 0.25 wt.% Sc and 0.08 wt.% Zr enabled this alloy to resist recrystallization even after 8 h at 520 °C. Similarly, an Al-5.8Mg-0.4Mn-0.25Sc-0.1Zr alloy recrystallized 150 °C higher than its Sc-free counterpart [16]. Lu et al. reported [13] that adding 0.14 wt.% Sc and 0.12 wt.% Zr to an Al-6Mg-0.4Mn alloy increased its recrystallization start temperature from 200 to 420 °C. Another study by Shen et al. [14] reported that the strength of Al-6.15Mg-0.3Sc-0.15Zr alloys dropped only at 500 °C due to the precipitate coarsening and reduced dislocation density caused by recovery and recrystallization. Alloy AA5182 with 0.1 wt.% Sc and 0.1 wt.% Zr alloy commenced partial recrystallization after 2 h at 400 °C, while the conventional AA5182 alloy already fully recrystallized after 2 h at 300 °C [3]. However, the influence of annealing temperature on the recrystallization behavior and microstructural evolution in Sc/Zr-containing AA5083 alloys has not been thoroughly investigated.

This study provides a comprehensive investigation into the influence of annealing temperature on the recrystallization behavior, microstructural evolution, and mechanical properties of cold-rolled AA5083 with a low Sc/Zr addition. While previous research has explored the effects of Sc/Zr additions on Al-Mg-Mn alloys, the current study is distinct in focusing on cost-effective, low-Sc alloying, aiming to balance performance enhancements with economic feasibility. By systematically correlating microhardness, grain structure, and precipitate behavior across a wide temperature range, this work addresses the knowledge gap in the recrystallization dynamics of AA5083 with a low Sc/Zr level. Moreover, the findings hold practical significance for designing lightweight, high-performance aluminum alloys for industrial applications, with improved strength and recrystallization resistance at an affordable cost.

## 2. Materials and Methods

**Figure 1:**
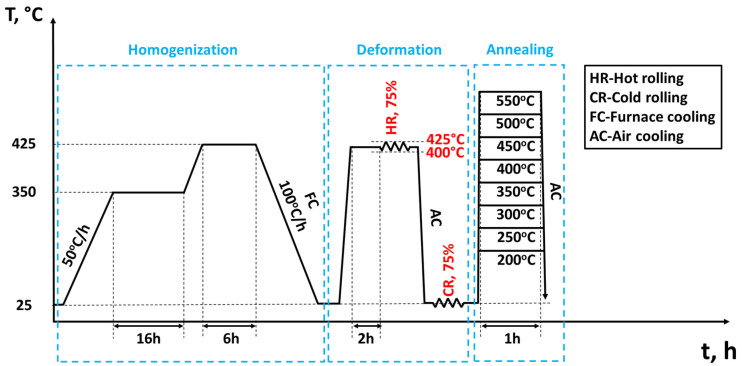
Thermomechanical processing route of AA5083 sheets.

Two typical Al-Mg-Mn alloys based on AA5083 were prepared: a Sc-free base alloy and a Sc-bearing alloy. The base alloy was a standard AlMg4.5Mn0.5 alloy with 0.2 wt.% Fe and minor Si and Cr additions (<0.1 wt.% each). The second alloy was microalloyed with 0.1 wt.% Sc and 0.08 wt.% Zr while keeping the other elements the same as in the base alloy. Both alloys were direct-chill-cast into rectangular ingots with dimensions of 590 mm wide × 185 mm thick × 950 mm long at Rio Tinto’s Arvida Research and Development Centre in Saguenay, Quebec. Test samples were cut from slices of the cast ingot and subjected to two-step homogenization (350 °C/16 h + 425 °C/6 h) with a heating rate of 50 °C/h, followed by furnace cooling. The primary objectives of the homogenization treatment included (1) dissolving low-melting eutectic phases, notably the β-AlMg phase; (2) minimizing the microsegregation of alloying elements; and (3) facilitating the precipitation of thermally stable dispersoids (i.e., Mn-dispersoids and Al_3_(Sc,Zr)) [1,15]. The homogenized samples were hot-rolled to 4 mm thickness at 400–425 °C in multi-passes with a total reduction of 75%. Subsequently, the hot-rolled sheets were cold-rolled to 1 mm thickness with a 75% reduction, followed by isothermal annealing at 200, 250, 300, 350, 400, 450, 500, and 550 °C for 1 h, respectively. The processing route of the materials is illustrated in Figure 1.

Samples for the hardness measurement and microstructural observation were prepared using a standard metallographic procedure to ensure a good polishing surface. The Vickers hardness of all annealed samples was measured using an NG-1000 CCD machine (NextGen Material Testing, Vancouver, BC, Canada) with a 25 g load and a 20 s dwell time. Twenty indentations were performed on the polished surface of each sample, and the average value was calculated. The microstructures under various processing conditions were observed using optical microscopy (Nikon, Eclipse ME600, Nikon Instruments Inc., Melville, NY, USA), scanning electron microscopy (SEM, JEOL-JSM-6480LV, JEOL USA Inc., Peabody, MA, USA), and transmission electron microscopy (TEM, JEOL-JEM-2100, JEOL USA Inc., Peabody, MA, USA). The grain structure was characterized under polarized light after electro-etching using Barker’s reagent (4 mL HBF_4_ + 200 mL H_2_O) at 17 V for 90 s. Grain structure analyses of annealed samples parallel to the rolling direction were performed using the electron backscatter diffraction technique (EBSD) on a sample area of 400 µm × 200 µm with a scan step size of 0.5 µm. Data obtained from the EBSD results were processed using the HKL Channel 5 software (version 5.0). Thin foils for TEM analysis were prepared in a twin-jet electro-polisher using a 30% nitric acid solution in methanol at 20 V and −20 °C. Bright- and dark-field images were captured near the [100] zone axis to examine the AlMn dispersoids and Al_3_(Sc,Zr) precipitates.

## 3. Results

### 3.1. Microhardness Evolution During Annealing

**Figure 2:**
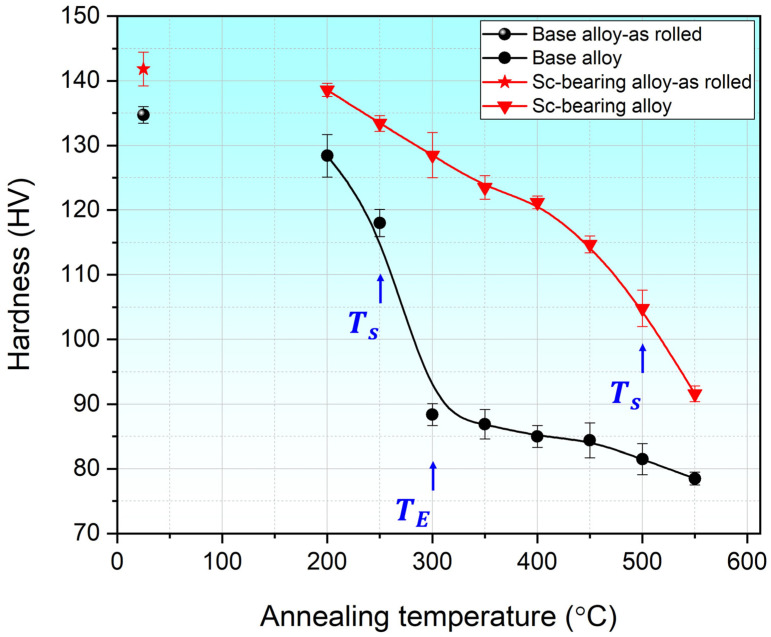
Hardness evolution during annealing at different temperatures.

Figure 2 shows the effect of Sc/Zr on the microhardness of cold-rolled AA5083 sheets as a function of annealing temperature. Initially, the as-cold-rolled samples of both alloys exhibited remarkably high hardness values due to the strain hardening from cold rolling. During annealing, the hardness began to decrease in both alloys, but the Sc-bearing alloy always maintained higher hardness values relative to the base alloy. For the base alloy, the hardness decreased slightly when annealing at 200 °C, followed by a sharp drop from 118 HV at 250 °C (start of static recrystallization, *T_s_*) to 88.4 HV at 300 °C (end of static recrystallization, *T_E_*), and then a slow decrease with increasing annealing temperature to 78.5 HV at 550 °C. In contrast, the Sc-bearing alloy showed a gradual decrease in hardness with increasing annealing temperature up to 500 °C. A notable drop in hardness then occurred from 105 HV at 500 °C to 92 HV at 550 °C, suggesting the possible beginning of recrystallization at 500 °C. It should be mentioned that the hardness of the Sc-bearing alloy consistently surpassed that of the base alloy across all annealing temperatures, demonstrating the strengthening effect of Al_3_(Sc,Zr) nanoparticles [1,10,18].

### 3.2. Grain Structure Evolution During Annealing

**Figure 3:**
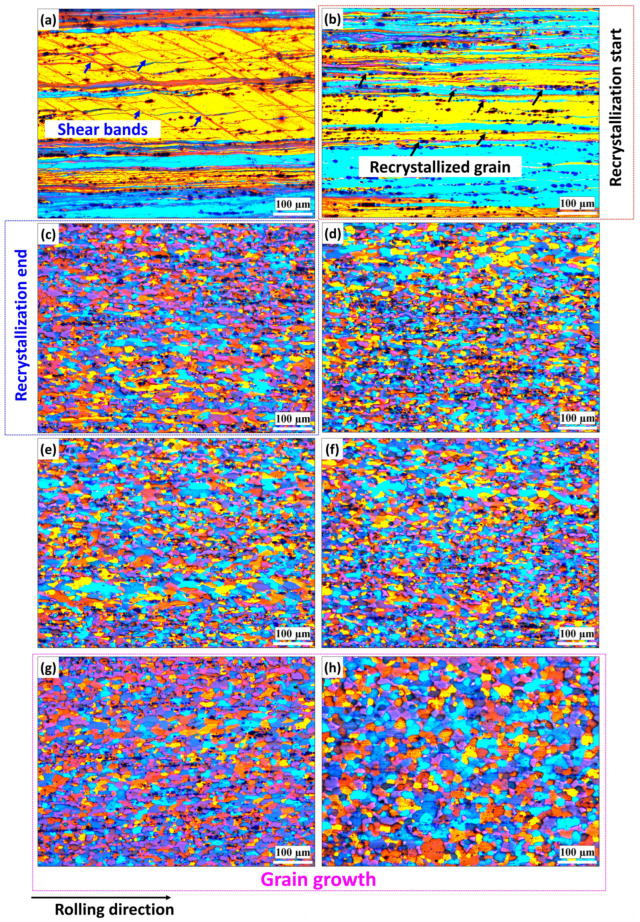
Representative polarized-light optical images after electrolytic etching showing grain structure evolution of the base alloy at various annealing temperatures: (**a**) 200 °C, (**b**) 250 °C, (**c**) 300 °C, (**d**) 350 °C, (**e**) 400 °C, (**f**) 450 °C, (**g**) 500 °C, and (**h**) 550 °C.

**Figure 4:**
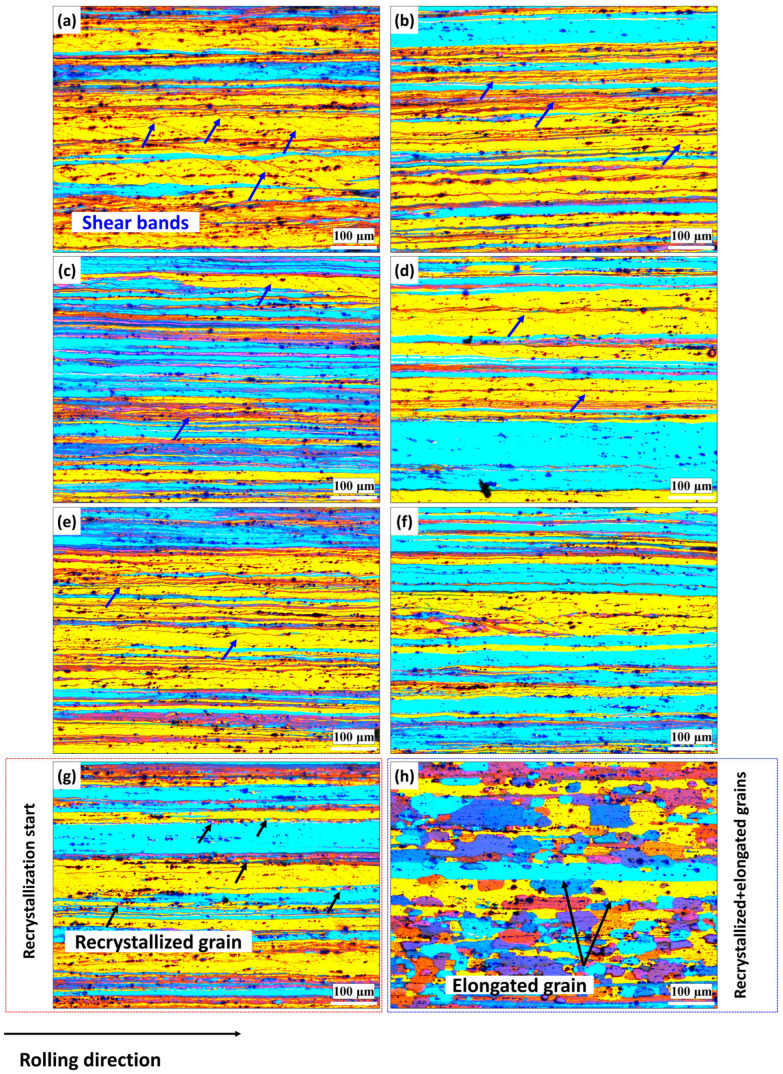
Representative polarized-light optical images showing grain structure evolution of the Sc-bearing alloy at various annealing temperatures: (**a**) 200 °C, (**b**) 250 °C, (**c**) 300 °C, (**d**) 350 °C, (**e**) 400 °C, (**f**) 450 °C, (**g**) 500 °C, and (**h**) 550 °C.

Figure 3 and Figure 4 depict the grain structure changes in both the base and Sc-bearing alloys during annealing, observed using polarized-light microscopy after electro-etching. For the base alloy, all grains exhibited a deformed structure in the rolling direction after annealing at 200 °C, with some shear bands shown with blue arrows in Figure 3a. At 250 °C, tiny grains appeared in the deformed structure (shown by black arrows), indicating that recrystallization started (Figure 3b). Full recrystallization with fine and equiaxed grains occurred when the annealing temperature reached 300 °C (Figure 3c). The size of the equiaxed grains remained stable until annealing at 450 °C (Figure 3d–f), mainly due to the pinning effect of Mn-bearing dispersoids [19]. Significant grain growth was observed when the annealing temperature increased to 500 and 550 °C, as shown in Figure 3g,h, respectively. On the other hand, the Sc-bearing alloys maintained the deformed grain structure along the rolling direction (Figure 4a–f) for up to 450 °C. Shear bands were clearly visible at low annealing temperatures but diminished as the annealing temperature increased. No substantial recrystallization was observed in the temperature range of 200–450 °C. However, at 500 °C, recrystallization started with the appearance of some fine grains within a deformed structure (Figure 4g, see black arrows). By 550 °C, the microstructure was not fully recrystallized yet and still displayed some elongated deformed grains aligned in the rolling direction between the recrystallized equiaxed grains (Figure 4h).

**Figure 5:**
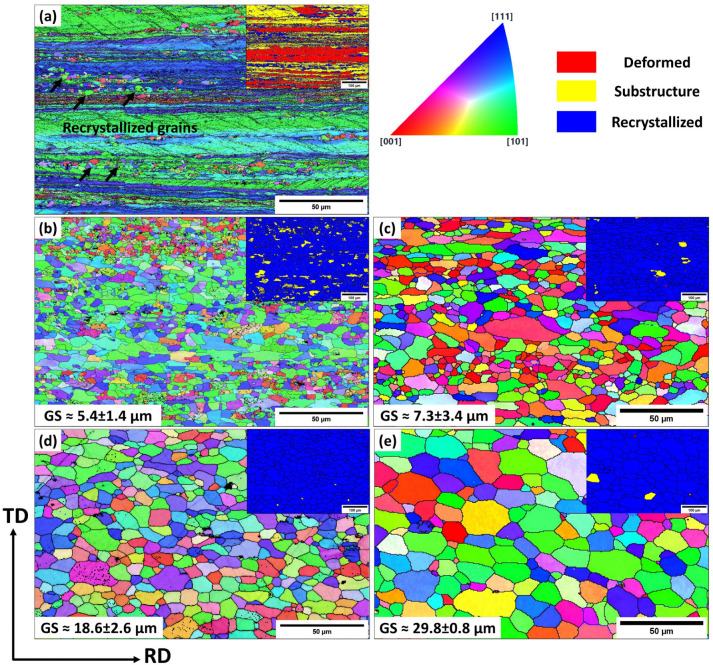
EBSD inverse-pole figure (IPF) and inserted grain orientation spread (GOS) maps of the base alloy annealed at (**a**) 250 °C, (**b**) 300 °C, (**c**) 450 °C, (**d**) 500 °C, and (**e**) 550 °C.

**Figure 6:**
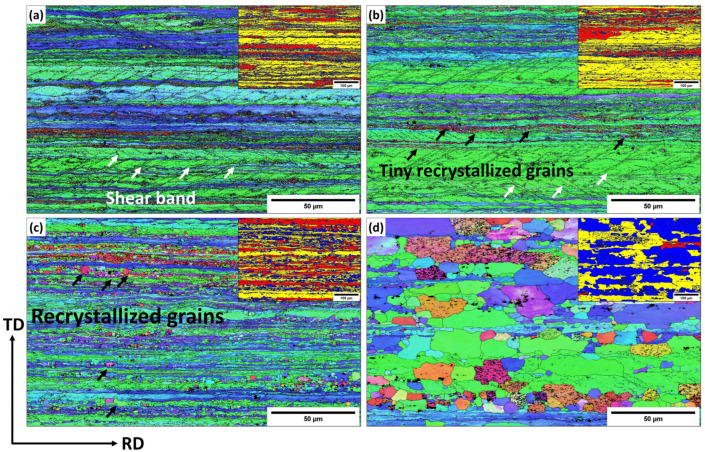
EBSD inverse-pole figure (IPF) and inserted grain orientation spread (GOS) maps of the Sc-bearing alloy annealed at (**a**) 300 °C, (**b**) 450 °C, (**c**) 500 °C, and (**d**) 550 °C.

**Figure 7:**
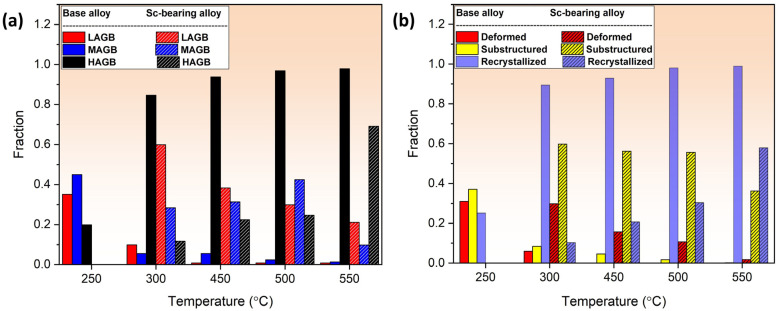
Statistical analysis of (**a**) the grain boundary misorientation distribution based on IPF maps and (**b**) grain structure distribution based on GOS maps.

In addition to the optical microscopy (OM) observations, a detailed analysis of grain structures on the annealed samples was conducted using the EBSD technique, which included inverse-pole figure (IPF) and grain orientation spread (GOS) maps (Figure 5 and Figure 6, respectively). Moreover, Figure 7 presents the statistical analysis of the EBSD maps, covering the grain boundary misorientation and recrystallization fractions according to the criteria applied in [20]. Grain orientation spread (GOS) indicates the average misorientations within grains, with blue for recrystallized (GOS < 2°), yellow for substructured (GOS 2–15°), and red for deformed (GOS > 15°) grains. The EBSD results are consistent with the OM observations in Figure 3 and Figure 4. For the base alloy, the initial recrystallization at 250 °C exhibited tiny, recrystallized grains (~0.5–2 µm) distributed between elongated deformed grains (Figure 5a). At 300 °C (Figure 5b), the recrystallization was mostly completed, as demonstrated by a predominantly equiaxed grain structure with an average grain size of 5.4 µm. Figure 7a shows that the fraction of high-angle grain boundaries (HAGBs) significantly increased from 40% at 250 °C to 85% at 300 °C, indicating accelerated static recrystallization as the annealing temperature increased from 250 to 300 °C. Consequently, the recrystallization fraction rose from 25% to 90% between these two temperatures (Figure 7b).

Recrystallized grains grew modestly until 450 °C with a growth rate of 35%, compared to those annealed at 300 °C, likely due to Mn-dispersoids hindering grain boundary movement. However, the grain growth accelerated at higher annealing temperatures (Figure 5c–e). At 500 and 550 °C, fast growth was observed with growth rates of 244% at 500 °C and 451% at 550 °C, respectively, mainly due to the coarsening of Mn-dispersoids at higher temperatures (Figure 5e) [19,21,22].

For the Sc-bearing alloy, the deformed grain structure remained stable up to 450 °C, with visible shear bands (white arrows) and a “necklace” pattern of tiny ~0.2–0.5 µm recrystallized grain nuclei (black arrows) appearing near the shear bands [23], as shown in Figure 6a,b. However, at 500 °C (Figure 6c), the start of recrystallization was identified by a number of fine equiaxed grains (1–2 µm) free of subgrain boundaries within the deformed structure, causing a notable drop in hardness at 500 °C (Figure 2). The GOS analysis (Figure 7b) showed a slow increase in the recrystallization fraction with increasing annealing temperature from 300 to 500 °C, in which the recrystallization fraction increased from 8% at 300 °C to 30% at 500 °C. When the temperature reached 550 °C, the microstructure was still not yet fully recrystallized, retaining some elongated deformed grains, as shown in Figure 6d. The fraction of HAGBs significantly increased from 23% at 500 °C to 69% at 550 °C with a high recrystallization fraction of 58%, as shown in Figure 7. The above results indicate that microalloying AA5083 with 0.1 wt.% Sc and 0.08 wt.% Zr effectively doubles the recrystallization temperature.

### 3.3. TEM Observations

**Figure 8:**
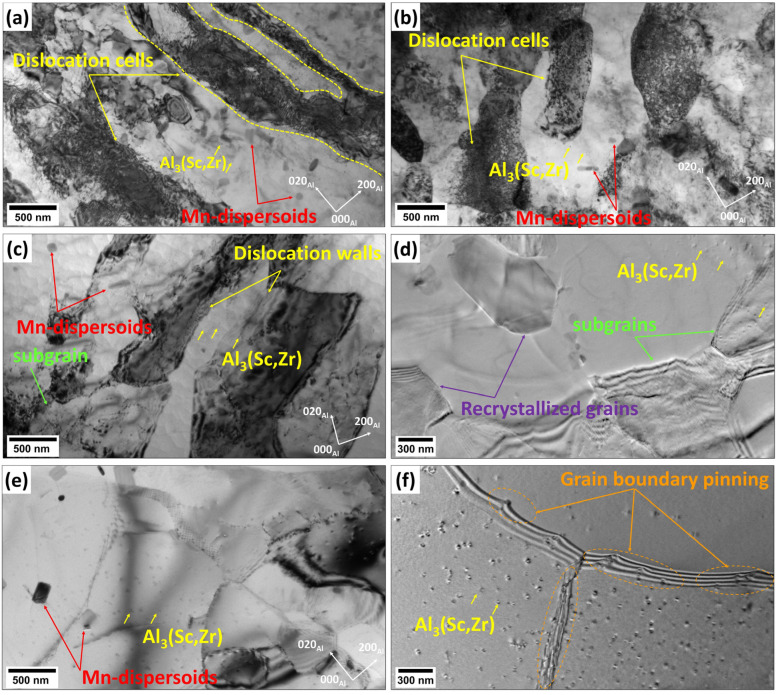
Bright-field TEM images of the Sc-bearing alloy annealed at (**a**) 300 °C, (**b**) 450 °C, (**c**,**d**) 500 °C, and (**e**,**f**) 550 °C.

The bright-field TEM micrographs in Figure 8 reveal distinct microstructural change in the Sc-bearing alloy at various annealing temperatures. At 300 °C, the alloy exhibited extensive dislocation cells with high dislocation densities spanning large areas, along with notable dislocation rearrangement near the grain boundaries, forming a pronounced cellular substructure (Figure 8a). Upon increasing the annealing temperature to 450 °C, the microstructure retained a high dislocation density, accompanied by the formation of subgrains with low-angle boundaries due to a polygonization process (Figure 8b). Additionally, a number of Mn-dispersoids and Al_3_(Sc,Zr) nanoparticles effectively pinned dislocations and subgrain migration, thus delaying the recrystallization process. At 500 °C, a remarkable decrease in the dislocation density coincided with an increase in the subgrain size and the emergence of well-defined subgrain structures (Figure 8c,d). Figure 8d provides an enlarged view of the recrystallization process at 500 °C, displaying the formation of new recrystallized grains, which exhibit a low dislocation density and are free of subgrain boundaries. At 550 °C, the subgrain coalescence and recrystallized grain growth were advanced (Figure 8e), with larger and more recrystallized grains compared to those at 500 °C (Figure 8c). This stage showed the progress of recrystallization, driven by high thermal energy, which facilitated grain boundary migration. However, thermally stable Al_3_(Sc,Zr) nanoparticles in the Al matrix still effectively pinned subgrain and grain boundaries (Figure 8f), slowing down the boundary migration and movement and stabilizing the microstructure during high-temperature annealing.

## 4. Discussion

### 4.1. Recrystallization Resistance

Figure 2 shows the different hardness responses with an increasing annealing temperature of both alloys, which reflects the different recrystallization behaviors between the two alloys. It is well known that the annealing of cold-rolled sheets softens and stabilizes the material and, hence, significantly affects its formability through three mechanisms: static recovery (SRV), static recrystallization (SRX), and grain growth. The degree of SRV and SRX depends on the annealing conditions (temperature and time) and the presence of secondary particles in the Al matrix [6,14,24]. SRV reduces the dislocation density and internal stress by rearranging and reducing dislocations within the material, transforming the deformed structure to a substructured grain structure, whereas SRX further eliminates internal stresses by forming new and strain-free grains. Increasing the annealing temperature facilitates dislocation rearrangement and annihilation, promoting the formation of subgrains and accelerating recrystallization kinetics [6,14,24]. Additionally, secondary dispersed particles delay recrystallization and hinder grain growth by pinning dislocation and boundary movements [17,25,26].

**Figure 9:**
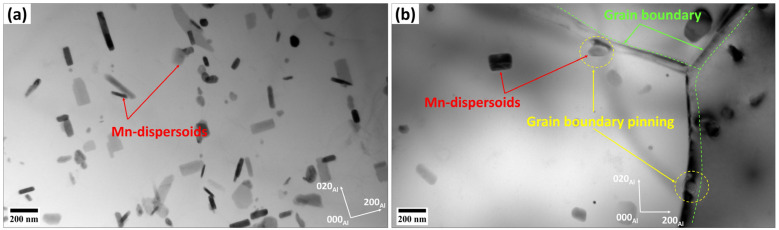
Bright-field TEM images of the base alloy showing (**a**) Mn-dispersoid distribution after homogenization and (**b**) the distribution and pinning effect of the Mn-dispersoids on the grain boundaries after annealing at 450 °C.

According to the results in Figure 3 and Figure 5, the base alloy underwent SRV up to 250 °C, primarily by dislocation rearrangements in the deformed structure. The SRX in the base alloy began at 250 °C and ended at 300 °C, accompanied by a sharp decrease in hardness (ΔHV_250–300°C_ = 30, Figure 2). Actually, both alloys contained Mn-dispersoids after homogenization, and Figure 9 shows an example of the Mn-dispersoid distribution in the base alloy. It seems that the Mn-dispersoids in the base alloy were insufficient to efficiently impede the recrystallization but could slow down grain growth after recrystallization from 300 to 500 °C.

**Figure 10:**
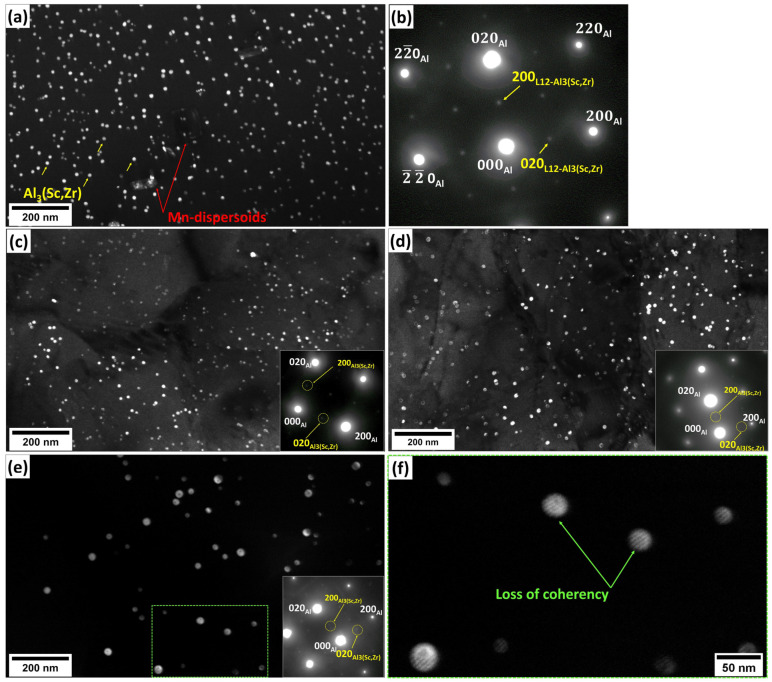
Dark-field TEM images of the Sc-bearing alloy showing Al_3_(Sc,Zr) evolution (**a**) during homogenization, with (**b**) the corresponding SAED pattern, and during annealing at (**c**) 450 °C, (**d**) 500 °C, and (**e**,**f**) 550 °C, respectively. (**e**) An enlarged image showing the coherency loss of Al_3_(Sc,Zr) at 550 °C.

In contrast, the Sc-bearing alloy continued the SRV up to 500 °C with a gradual hardness decrease (ΔHV_200–500°C_ = 34) and started SRX from 500 to 550 °C (Figure 4 and Figure 6), raising the starting temperature of recrystallization to 500 °C, which was 250 °C higher than that of the base alloy. During homogenization, the Sc-bearing alloy precipitated a large number of fine Al_3_(Sc,Zr) nanoparticles along with Mn-dispersoids. The dark-field TEM images in Figure 10 display the distribution of these Al_3_(Sc,Zr) precipitates under different homogenization and annealing conditions, and Table 1 provides the quantitative results of the particle characteristics. The Al_3_(Sc,Zr) nanoparticles remained relatively stable during annealing up to 500 °C, with a slight decrease in number density and a slight increase in size. However, those nanoparticles coarsened and lost their coherence at 550 °C [27,28,29,30]. It is obvious that the presence of nanosized Al_3_(Sc,Zr) precipitates in the Sc-bearing alloy delayed recrystallization to higher temperatures.

The classical Zener drag pressure is widely used to describe the recrystallization behavior of a material in the presence of secondary particles, which exerts a delaying pressure on the moving grain boundaries during recrystallization and can be expressed as follows [20,31,32].

(1) $$P_{z}=\frac{3\gamma_{GB}f_{v}}{\bar{D}}\;,\;$$

where $P_{z}$ is the Zener drag pressure, $f_{v}$ and $\bar{D}\;$ are the volume fraction and average equivalent diameter of the secondary particles, and $\gamma_{GB}$ is the specific grain boundary energy, which is typically 0.3 J·m^−2^ for Al alloys [33]. The calculated $P_{z}$ values induced by individual secondary particles are listed in Table 1.

Owing to their large size and low number density, the pinning effect of Mn-dispersoids is weak, and the $P_{z}$ is low. Because of severe cold rolling with a reduction of 75% in this study, the cold-rolled sheets in both alloys had a considerably high stored strain energy. Therefore, recrystallization in the base alloy started at a rather low temperature and was fully completed within a short temperature interval (from 250 to 300 °C), showing a low recrystallization resistance. On the other hand, the Sc-bearing alloy consisted of a high number density of small Al_3_(Sc,Zr) nanoparticles, and therefore, the $P_{z}$ induced by Al_3_(Sc,Zr) was significantly higher than that of Mn-dispersoids. It appears that the Al_3_(Sc,Zr) nanoparticles alongside the Mn-dispersoids in the Sc-bearing alloy could efficiently exert a sufficient pinning effect on the motion of dislocations and grain/subgrain boundaries so as to maintain SRV up to 500 °C despite the high strain energy in the material. This was also explained by the fact that the $P_{z}$ of Al_3_(Sc,Zr) in the Sc-containing alloy only slightly decreased from the as-homogenized state to the 500 °C annealed state. However, the $P_{z}$ then sharply dropped from 500 to 550 °C, which facilitated the dislocation slip and grain boundary motion, resulting in the recrystallization that occurred at 500–550 °C. Even with such high-temperature annealing, the Al_3_(Sc,Zr) nanoparticles still hindered the recrystallization process. The fact that the Sc-bearing alloy was only 58% recrystallized after annealing at 550 °C demonstrated a much stronger recrystallization resistance relative to the base alloy.

### 4.2. Boundary Strengthening Contribution

As described in Figure 3, Figure 4, Figure 5, Figure 6 and Figure 7, the grain structure changed significantly with increasing annealing temperature owing to SRV and SRX processes accompanied by large changes in the fractions of LAGBs and HAGBs, thereby reducing the mechanical strength of the rolled sheets. Additionally, the presence of nanosized Al_3_(Sc,Zr) precipitates in the Sc-bearing alloy strongly influenced the grain structure during annealing, and therefore, the two alloys exhibited different grain structures and recrystallization behaviors. To understand the influence of grain boundary strengthening on the yield strength (YS) during annealing, the strengthening contribution from grain boundaries ($∆\sigma_{GB}$) could be estimated using the following equation [34,35]:

(2) $$\begin{array}{ll} ∆\sigma_{GB} & =\sigma_{HAGB}+\sigma_{LAGB}\\ & =(\frac{K_{y}}{{{(d}_{HAGB})}^{0.5}}){\left(f_{HAGB}\right)}^{0.5}+M\alpha G{\left[1.5bS_{v}\theta_{ave}^{LAGB}f_{LAGB}\right]}^{0.5} \end{array}$$

where $K_{y}$ is the Hall–Petch constant (=105 MPa·μm^−0.5^) [33,34], and $d_{HAGB}$ is the average grain size of recrystallized grains with HAGBs, which was measured based on the EBSD images (Figure 8d–f) using the intercept method according to ASTM E112-12 [36]. Here, *M* is the Taylor factor with a value of 3, *α* is a constant taken to equal 0.24 [33], *G* is the shear modulus of Al at ambient temperature with a value of 27.4 GPa [1,37], and *b* is the Burgers vector with a value of 0.286 nm [35]. $S_{v}=(\frac{4}{\pi }B_{A})$, where *B_A_* is the total boundary length per unit area (from the 2-D micrographs). $S_{v}=(\frac{4}{\pi }B_{A})$ and $f_{LAGB}$ are the average misorientation angle and the fraction of the LAGBs, respectively, and the cutoff angle of LAGBs was chosen to be 15° [34].

The parameters $d_{HAGB}$, $S_{v}$, $\theta_{ave}^{LAGB},$ and $f_{LAGB}$, derived from the EBSD results (Figure 5, Figure 6 and Figure 7) are listed in Table 2. By applying those parameters to Equation (2), the YS contributions of grain boundary strengthening, $∆\sigma_{GB}$, during annealing were calculated. As shown in Table 2, with increasing annealing temperature, $∆\sigma_{GB}$ became smaller owing to increased SRV and SRX in both alloys. However, $∆\sigma_{GB}$ in the Sc-bearing alloy was far superior to that in the base alloy, because of the presence of numerous Al_3_(Sc,Zr) nanoprecipitates.

**Figure 11:**
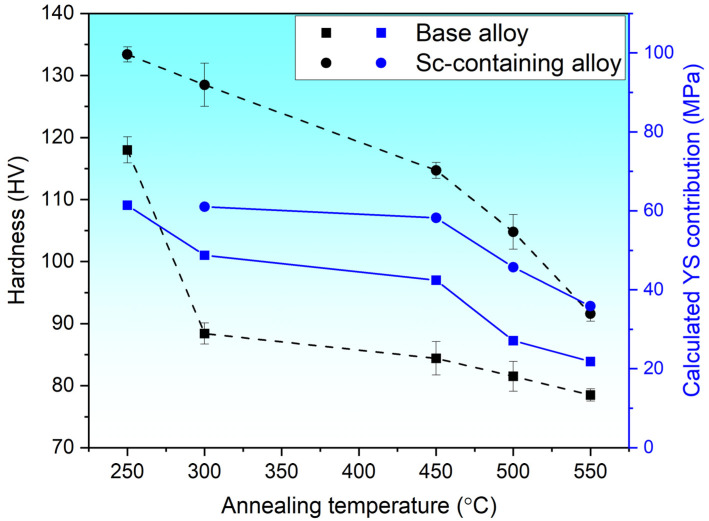
Experimentally measured hardness and calculated YS contribution from grain boundaries as a function of annealing temperature.

Figure 11 presents the relationship between the boundary strength contribution $∆\sigma_{GB}$ and the measured hardness as a function of annealing temperature for both alloys. Notably, $∆\sigma_{GB}$ exhibited a similar trend as the hardness with increasing temperature. The base alloy displayed a significant drop in grain boundary strengthening from 61.4 MPa at 250 °C to 48.7 MPa at 300 °C, while the hardness decreased remarkably from 118 HV at 250 °C to 88 HV at 300 °C. The decrease in $∆\sigma_{GB}$ from 450 °C to 550 °C also mirrors the trend of decreasing hardness with increasing annealing temperature. Meanwhile, the Sc-bearing alloy showed a steady decline in $∆\sigma_{GB}$ from 60.8 MPa at 300 °C to 45.7 MPa at 500 °C and further to 35.9 MPa at 550 °C, whereas the hardness showed a similar tendency as it gradually decreased from 128 HV at 300 °C to 105 HV at 500 °C and further to 92 HV at 550 °C. These findings imply that the grain structure change during annealing and the corresponding grain boundary strengthening have a profound impact on the mechanical strength.

The mechanical strength of annealed materials is mainly influenced by secondary particle strengthening and grain boundary strengthening. Both the base and Sc-bearing alloys contained Mn-dispersoids. However, because of their low number density and relatively large size, the particle strengthening from Mn-dispersoids was relatively weak. In contrast, the particle strengthening from Al_3_(Sc,Zr) precipitates in the Sc-bearing alloy was strong due to their much higher number density and finer size. Therefore, the mechanical strength (as measured in terms of hardness) of the Sc-bearing alloy was always higher relative to the base alloy. This was particularly evident at the low annealing temperature where both alloys had the same fully deformed gain structure. However, for a given annealed condition, the grain boundary strengthening was an important factor determining the alloy strength, as suggested by the fact that $∆\sigma_{GB}$ and hardness showed similar tendencies with the annealing temperature. This was also because both secondary particles (Mn-dispersoids and Al_3_(Sc,Zr) precipitates) were relatively thermally stable up to about 500 °C, and their size and number density remained almost unchanged during short annealing treatments. When the Mn-dispersoids and Al_3_(Sc,Zr) precipitates became unstable and coarsened above 500 °C, the decreasing strengthening effects from particle coarsening and grain growth acted together to accelerate the decline in strength.

## 5. Conclusions

The mechanical properties, microstructural evolution, and recrystallization behavior of AA5083-based cold-rolled sheets with and without Sc/Zr microalloying were systematically studied as a function of annealing temperatures. The main findings are summarized as follows:

1. During annealing, the hardness of the base alloy significantly decreased from 118 HV at 250 °C to 88.4 HV at 300 °C, indicating that static recrystallization occurred at 250–300 °C. In contrast, the Sc-bearing alloy showed a gradual decrease in hardness with increasing annealing temperature up to 500 °C, suggesting that static recrystallization commenced at about 500 °C. The hardness of the Sc-bearing alloy consistently surpassed that of the base alloy at all annealing temperatures.
2. A minor addition of 0.1 wt.%Sc and 0.08 wt.% Zr improved the alloy strength and recrystallization resistance. The recrystallization temperature was effectively doubled from 250 °C for the base alloy to 500 °C for the Sc-bearing alloy.
3. The addition of Sc/Zr to AA5083 generated a high number density of nano-sized Al_3_(Sc,Zr) precipitates. The increased recrystallization resistance of the Sc-bearing alloy was mainly attributed to the presence of those nanoparticles, which enhanced the Zener drag pressure and delayed recrystallization due to their strong pinning effects on dislocations and grain/subgrain boundaries.
4. The grain boundary strengthening effects at various annealing temperatures were estimated using a constitutive equation. The calculated yield strength and measured hardness values exhibited similar trends with the annealing temperature, implying that the grain structure change and corresponding grain boundary strengthening effect were predominant factors controlling the alloy’s strength evolution during annealing.

## Figures and Tables

**Table 1:** Characteristics of secondary particles during homogenization and annealing treatments.

Alloy	Condition	Equivalent Diameter (D), nm	Number Density, $\left(N_{d}\right)$, m^−3^	Volume Fraction, $\left(V_{f}\right),\%$	$P_{z}$ of Particles, MPa
Base alloy for Mn-dispersoids	As-Homo	63.4 ± 3.4	1.5 ± 0.8 × 10^20^	0.74	**0.099**
Sc-bearing alloy for Al_3_(Sc,Zr) precipitates	As-Homo	13.5 ± 0.8	5.1 ± 0.1 × 10^21^	0.479	**0.329**
	450 °C	13.5 ± 1.4	5.0 ± 0.4 × 10^21^	0.476	**0.320**
	500 °C	14.2 ± 1.1	4.3 ± 0.8 × 10^21^	0.454	**0.288**
	550 °C	30.2 ± 0.9	4.1 ± 1.2 × 10^20^	0.196	**0.058**

**Table 2:** Measured grain boundary parameters based on the EBSD results and the corresponding strengthening contributions for the two alloys studied.

Ann. Temp., (°C)	Alloy	$d_{HAGs}$, (µm)	$\theta_{ave}^{LAGB}$, (°)	$f_{LAGB}$, %	$S_{v}$	$∆\sigma_{GB}$ (MPa)
**250**	base	0.96	5.33	85.1	0.53	**61.4**
	Sc-bearing	---	---	---	---	**---**
**300**	base	5.41	7.32	15.4	0.28	**48.7**
	Sc-bearing	0.95	5.98	88.3	0.65	**60.8**
**450**	base	7.30	8.41	6.3	0.26	**42.4**
	Sc-bearing	1.90	5.13	72.3	0.53	**58.2**
**500**	base	18.60	10.61	3.1	0.18	**27.1**
	Sc-bearing	3.86	5.05	69.6	0.45	**45.7**
**550**	base	29.20	12.11	2.1	0.16	**21.8**
	Sc-bearing	10.46	4.86	30.9	0.32	**35.9**

## Data Availability

The original contributions presented in this study are included in the article. Further inquiries can be directed to the corresponding author.

## References

[1] Algendy A.Y., Liu K., Rometsch P., Parson N., Chen X.-G. (2022). Effects of AlMn dispersoids and Al_3_ (Sc, Zr) precipitates on the microstructure and ambient/elevated-temperature mechanical properties of hot-rolled AA5083 alloys. Mater. Sci. Eng. A.

[2] Pan S., Wang Z., Li C., Wan D., Chen X., Chen K., Li Y. (2023). Achieving superior dispersion-strengthening effect in an AA5xxx Al-Mg-Mn alloy by mico-alloying. Mater. Des..

[3] Qiu Y., Yang X., Li J., Xiang S., Shi J., Xu J., Sanders R.E. (2022). The influence of Sc and Zr additions on microstructure and corrosion behavior of AA5182 alloy sheet. Corros. Sci..

[4] Deng Y., Zhang G., Yang Z., Xu G. (2019). Microstructure characteristics and mechanical properties of new aerospace Al-Mg-Mn alloys with Al_3_ (Sc1—xZrx) or Al_3_ (Er1—xZrx) nanoparticles. Mater. Charact..

[5] Li Z., Zhang Z., Chen X.-G. (2018). Improvement in the mechanical properties and creep resistance of Al-Mn-Mg 3004 alloy with Sc and Zr addition. Mater. Sci. Eng. A.

[6] Tang Z., Jiang F., Long M., Jiang J., Liu H., Tong M. (2020). Effect of annealing temperature on microstructure, mechanical properties and corrosion behavior of Al-Mg-Mn-Sc-Zr alloy. Appl. Surf. Sci..

[7] Dorin T., Ramajayam M., Vahid A., Langan T. (2018). Aluminium Scandium Alloys, Fundamentals of Aluminium Metallurgy.

[8] Guo Z., Zhao G., Chen X.-G. (2015). Effects of two-step homogenization on precipitation behavior of Al_3_Zr dispersoids and recrystallization resistance in 7150 aluminum alloy. Mater. Charact..

[9] Sun F., Nash G.L., Li Q., Liu E., He C., Shi C., Zhao N. (2017). Effect of Sc and Zr additions on microstructures and corrosion behavior of Al-Cu-Mg-Sc-Zr alloys. J. Mater. Sci. Technol..

[10] Wang Y., Liu H., Ma X., Wu R., Sun J., Hou L., Zhang J., Li X., Zhang M. (2019). Effects of Sc and Zr on microstructure and properties of 1420 aluminum alloy. Mater. Charact..

[11] Humphreys F.J., Hatherly M. (2012). Recrystallization and Related Annealing Phenomena.

[12] Bai Z.H., Luo B.H. (2011). The Effects of Annealing Temperature on the Microstructure and Properties of Cold-Rolled 5083 Aluminum Alloy. Adv. Mater. Res..

[13] Lu L., Jiang F., Liu J., Zhang J., Wang G., Feng B., Tong M., Tang Z. (2020). The influence of annealing temperature on microstructure, mechanical properties, and corrosion resistance of Al-6Mg-0.4 Mn-0.14 Sc-0.12 Zr alloy cold rolling plate. Front. Mater..

[14] Shen J., Chen B., Wan J., Shen J., Li J. (2022). Effect of annealing on microstructure and mechanical properties of an Al–Mg-Sc-Zr alloy. Mater. Sci. Eng. A.

[15] Rometsch P., Fourmann J., Elgallad E., Chen X.-G. (2023). Use of Sc to Improve the Properties of AA5083 Cast and Rolled Products. Proceedings of the TMS Annual Meeting & Exhibition; Light Metals 2023.

[16] Chen Q., Pan Q.-L., Wang Y., Zahng Z.-Y., Zhou J., Liu C. (2012). Microstructure and mechanical properties of Al-5.8 Mg-Mn-Sc-Zr alloy after annealing treatment. J. Cent. South Univ..

[17] Ocenasek V., Slamova M. (2001). Resistance to recrystallization due to Sc and Zr addition to Al–Mg alloys. Mater. Charact..

[18] Ikeshita S., Strodahs A., Saghi Z., Yamada K., Burdet P., Hata S., Ikeda K.-I., Midgley P.A., Kaneko K. (2016). Hardness and microstructural variation of Al–Mg–Mn–Sc–Zr alloy. Micron.

[19] Algendy A.Y., Liu K., Chen X.G. (2021). Evolution of dispersoids during multistep heat treatments and their effect on rolling performance in an Al-5% Mg-0.8% Mn alloy. Mater. Charact..

[20] Algendy A.Y., Rometsch P., Chen X.-G. (2024). Impact of hot rolling temperature on the mechanical properties and microstructural evolution of hot/cold-rolled AA5083 with Sc and Zr microalloying. Mater. Sci. Eng. A.

[21] Li C., Liu K., Chen X.-G. (2020). Improvement of elevated-temperature strength and recrystallization resistance via Mn-containing dispersoid strengthening in Al-Mg-Si 6082 alloys. J. Mater. Sci. Technol..

[22] Liu K., Chen X.G., Al-Mn-Mg D.O. (2015). 3004 alloy for applications at elevated temperature via dispersoid strengthening. Mater. Des..

[23] Huang H., Jiang F., Zhou J., Wei L., Qu J., Liu L. (2015). Effects of Al 3 (Sc, Zr) and Shear Band Formation on the Tensile Properties and Fracture Behavior of Al-Mg-Sc-Zr Alloy. J. Mater. Eng. Perform..

[24] Lee Y.-C., Tezuka H., Kobayashi E., Sato T. (2016). Effects of Annealing Temperature on the Recrystallization Behavior and Microstructure of Al-Mn Alloys with Different Second Phase Particles. Proceedings of the 8th Pacific Rim International Congress on Advanced Materials and Processing.

[25] Iwamura S., Nakayama M., Miura Y. (2002). Coherency Between Al_3_Sc Precipitate and the Matrix in Al Alloys Containing Sc. Materials Science Forum.

[26] Tang B.-Y., Li D.-L., Chen P., Yi J.-X., Wen L., Peng L.-M., Ding W.-J. (2010). The thermal properties of Al–Mg–TM (TM= Sc, Zr): Ab initio study. Solid State Sci..

[27] Fuller C.B., Seidman D.N. (2005). Temporal evolution of the nanostructure of Al(Sc,Zr) alloys: Part II-coarsening of Al_3_(Sc1-xZrx) precipitates. Acta Mater..

[28] Marquis E.A., Seidman D.N. (2005). Coarsening kinetics of nanoscale Al_3_Sc precipitates in an Al–Mg–Sc alloy. Acta Mater..

[29] Xu P., Jiang F., Tang Z., Yan N., Jiang J., Xu X., Peng Y. (2019). Coarsening of Al_3_Sc precipitates in Al-Mg-Sc alloys. J. Alloys Compd..

[30] Algendy A.Y., Rometsch P., Chen X.-G. (2025). Effects of Sc/Zr and Thermomechanical Processing on the Microstructure and Properties of AA5083 Rolled Products. Proceedings of the TMS Annual Meeting & Exhibition, Light Metals 2025.

[31] Nes E., Ryum N., Hunderi O. (1985). On the Zener. Acta Mater..

[32] Huang K., Marthinsen K., Zhao Q., Logé R.E. (2018). The double-edge effect of second-phase particles on the recrystallization behaviour and associated mechanical properties of metallic materials. Prog. Mater. Sci..

[33] Engler O., Liu Z., Kuhnke K. (2013). Impact of homogenization on particles in the Al-Mg-Mn alloy AA 5454-Experiment and simulation. J. Alloys Compd..

[34] Zha M., Tian T., Jia H.-L., Zhang H.-M., Wang H.-Y. (2023). Sc/Zr ratio-dependent mechanisms of strength evolution and microstructural thermal stability of multi-scale hetero-structured Al–Mg–Sc–Zr alloys. J. Mater. Sci. Technol..

[35] Su Y., Ding L., Zhang Y., Weng Y., Wang C., Jia Z., Zhuang L. (2025). The dispersoid evolution, recrystallization and mechanical properties of an Al–Mg–Sc alloy under various homogenization and annealing processes. J. Mater. Sci..

[36] (2012). Standard Test Methods for Determining Average Grain Size.

[37] Wang H., Geng H., Zhou D., Niitsu K., Muránsky O., Zhang D. (2020). Multiple strengthening mechanisms in high strength ultrafine-grained Al–Mg alloys. Mater. Sci. Eng. A.

